# Differential effects of P2Y_1_ deletion on glial activation and survival of photoreceptors and amacrine cells in the ischemic mouse retina

**DOI:** 10.1038/cddis.2014.317

**Published:** 2014-07-31

**Authors:** T Pannicke, I Frommherz, B Biedermann, L Wagner, K Sauer, E Ulbricht, W Härtig, U Krügel, U Ueberham, T Arendt, P Illes, A Bringmann, A Reichenbach, A Grosche

**Affiliations:** 1 Paul-Flechsig-Institut für Hirnforschung, Universität Leipzig, Leipzig, Germany; 2 Biotechnology Center, Technische Universität Dresden, Dresden, Germany; 3 Rudolf-Boehm-Institut für Pharmakologie und Toxikologie, Universität Leipzig, Leipzig, Germany; 4 Klinik und Poliklinik für Augenheilkunde, Universität Leipzig, Leipzig, Germany; 5 Institut für Humangenetik, Universität Regensburg, Regensburg, Germany

## Abstract

Gliosis of retinal Müller glial cells may have both beneficial and detrimental effects on neurons. To investigate the role of purinergic signaling in ischemia-induced reactive gliosis, transient retinal ischemia was evoked by elevation of the intraocular pressure in wild-type (Wt) mice and in mice deficient in the glia-specific nucleotide receptor P2Y_1_ (P2Y_1_ receptor-deficient (P2Y1R-KO)). While control retinae of P2Y1R-KO mice displayed reduced cell numbers in the ganglion cell and inner nuclear layers, ischemia induced apoptotic death of cells in all retinal layers in both, Wt and P2Y1R-KO mice, but the damage especially on photoreceptors was more pronounced in retinae of P2Y1R-KO mice. In contrast, gene expression profiling and histological data suggest an increased survival of amacrine cells in the postischemic retina of P2Y1R-KO mice. Interestingly, measuring the ischemia-induced downregulation of inwardly rectifying potassium channel (Kir)-mediated K^+^ currents as an indicator, reactive Müller cell gliosis was found to be weaker in P2Y1R-KO (current amplitude decreased by 18%) than in Wt mice (decrease by 68%). The inner retina harbors those neurons generating action potentials, which strongly rely on an intact ion homeostasis. This may explain why especially these cells appear to benefit from the preserved Kir4.1 expression in Müller cells, which should allow them to keep up their function in the context of spatial buffering of potassium. Especially under ischemic conditions, maintenance of this Müller cell function may dampen cytotoxic neuronal hyperexcitation and subsequent neuronal cell loss. In sum, we found that purinergic signaling modulates the gliotic activation pattern of Müller glia and lack of P2Y_1_ has janus-faced effects. In the end, the differential effects of a disrupted P2Y_1_ signaling onto neuronal survival in the ischemic retina call the putative therapeutical use of P2Y_1_-antagonists into question.

Glial cells are crucially involved in the maintenance of neuronal activity in nervous tissues.^1^ The homeostasis of the extracellular space is regulated by various glial functions including spatial K^+^ buffering, cell volume regulation and uptake of neurotransmitters.^2, 3, 4^ Activation of membrane receptors and ion channels is critically implicated in mediating the neuron-supportive glial functions. The dominant K^+^ conductance of glial cells mediates spatial K^+^ buffering and is important for the very negative membrane potential of these cells, thereby supporting electrogenic membrane transporters.^5^ Alterations in glial function are characteristic for pathological processes of the nervous system.^6^ Reactive gliosis may have beneficial and detrimental effects and is considered as an attempt to maintain neuronal function, protecting the tissue from further destruction, and to initiate tissue regeneration.^7, 8^ However, reactive gliosis may cause secondary neuronal damage as major neuron-supportive functions of glial cells get lost.^6^

Gliotic alterations of Müller cells, the dominant macroglia of the vertebrate retina, have been observed in various models of retinal diseases.^9, 10^ A prominent feature of Müller cell gliosis is the downregulation of the inwardly rectifying K^+^ conductance mediated by inwardly rectifying K^+^ (Kir) channels.^9^ It has been demonstrated in astrocytes that downregulation or conditional knockout of Kir4.1 results in an impairment of glial glutamate (Glu) uptake.^11, 12^ In addition, it has been suggested that autocrine/paracrine purinergic signaling may have a causative role in the development of reactive gliosis in brain and retina.^13, 14^ Müller cells express different subtypes of P2 nucleotide receptors including P2Y_1_ and P2Y_4_.^15, 16^ P2Y_1_ receptors have been demonstrated to be functionally expressed by Müller cells and microglial cells, rather than by neurons.^15, 16, 17, 18^

Retinal ischemia, a characteristic of various important human blinding diseases including diabetic retinopathy, results in neuronal degeneration and reactive gliosis.^19, 20^ The reduced K^+^ permeability of Müller cell membranes is associated with an impaired cell volume regulation under hypoosmotic stress after high intraocular pressure (HIOP)-induced ischemia.^21^ It has been observed that tandem-pore domain K^+^ channels may fulfill certain functions under conditions where Kir channels are downregulated or lacking.^22, 23^ A malfunctional Müller cell volume regulation was also found after deletion of P2Y_1_ in the mouse retina.^16^ It has been suggested that impaired glial K^+^ buffering and cell volume regulation may contribute to neuronal degeneration in the ischemic retina by inducing neuronal hyperexcitation and Glu-induced cell death.^14^ In order to determine whether endogenous purinergic signaling is implicated in mediating and/or protecting from neuronal degeneration, we investigated the effects of HIOP-induced ischemia in the retinae of P2Y_1_-deficient mice.

## Results

### Retinal morphology

**Figure 1:**
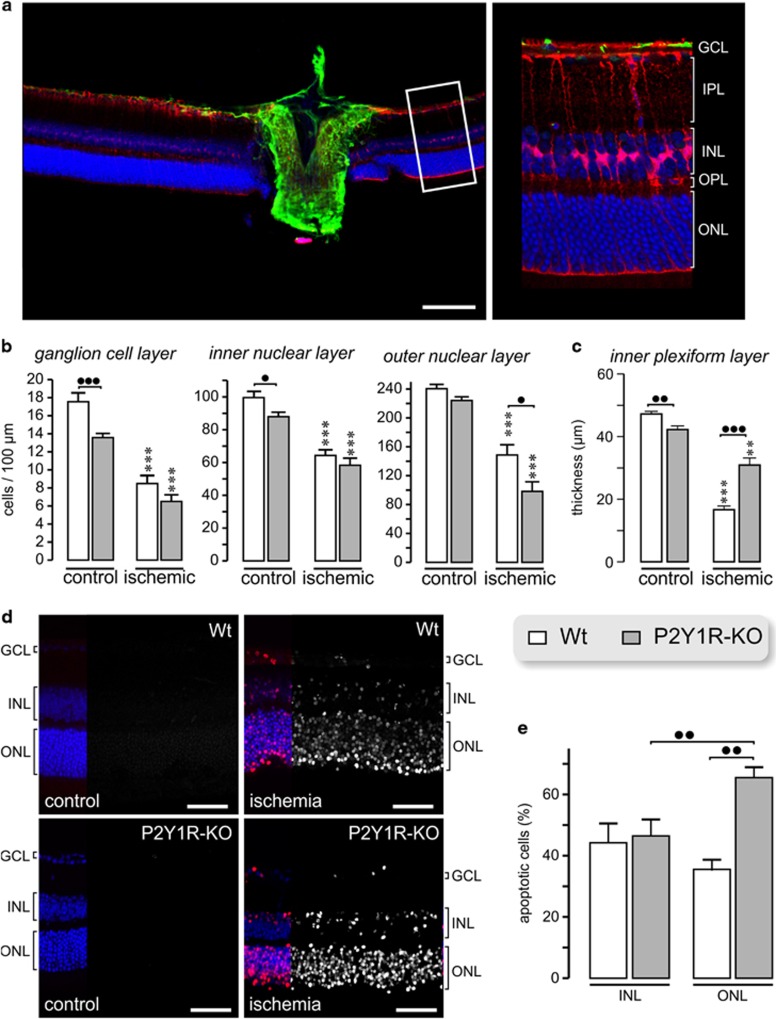
Ischemic degeneration of the murine retina after transient retinal ischemia of 90 min. (**a**) Counting of cell nuclei was performed in 100-*μ*m-wide areas close to the optic nerve head from retinae after 7 days survival — marked as the white square left and shown at larger magnification right. Cell nuclei were labeled with TO-PRO-3 (blue), astrocytes were immunostained with anti-GFAP antibodies (green) and Müller cells with anti-CRALBP antibodies (red).OPL, outer plexiform layer. (**b**) Mean±S.E.M. number of cell nuclei in different retinal layers. (**c**) Mean±S.E.M. thickness of the IPL. (**b** and **c**) Significant differences compared with the respective control eyes of the same genotype: ***P<0.001, **P<0.01. Significant differences between the indicated strains: ^●^*P*<0.05, ^●●^*P*<0.01, ^●●●^*P*<0.001. Data were obtained from 4 to 6 animals. From each animal, 4–6 central slices were analyzed leading to *n*=17–33. (**d**) Apoptotic cell death determined with TUNEL staining 1 day after transient retinal ischemia of 90 min. Staining of all cell nuclei (blue in the left parts of the images) and of TUNEL-labeled nuclei (red in the left parts and white in the right parts of the images) in retinae from Wt and P2Y1R-KO mice. Bars, 50 *μ*m. (**e**) Mean±S.E.M. number of TUNEL-labeled cell nuclei in the INL and ONL. ^●●^*P*<0.01. Apoptotic activity was determined in 13–18 central retinal slices derived from 4 to 5 animals

In order to compare the cell numbers between retinae from Wt and P2Y_1_ receptor-deficient (P2Y1R-KO) mice, we counted cell nuclei in the ganglion cell layer (GCL), inner nuclear layer (INL) and outer nuclear layer (ONL) in TO-PRO-3-labeled retinal slices from untreated eyes within a 100-*μ*m-wide area close to the optic nerve head (Figure 1a). As shown in Figure 1b, the retina of P2Y1R-KO mice displayed significantly reduced numbers of cell nuclei in the GCL and INL as compared with the Wt control retina. Moreover, the inner plexiform layer (IPL) of untreated P2Y1R-KO mice was significantly thinner than the IPL of Wt animals (Figure 1c).

Retinal ischemia-reperfusion results in neuronal degeneration.^19, 20^ In order to determine ischemic retinal degeneration, we induced transient retinal ischemia in mice for 90 min and quantified the number of cell nuclei in retinal slices 7 days after reperfusion. Transient ischemia caused significant reductions in the numbers of cell nuclei in retinae of Wt and P2Y1R-KO mice compared with untreated controls (Figure 1b). Interestingly, the number of cell (photoreceptor) nuclei in the ONL was more reduced (*P*<0.05) in P2Y1R-KO mice than in Wt retinae (Figure 1b). Although the thickness of the IPL was significantly reduced in the postischemic retinae of Wt and P2Y1R-KO mice, the decrease in the thickness of the IPL was significantly less pronounced in P2Y1R-KO mice (Figure 1c).

Apoptotic cell death was determined with terminal deoxynucleotidyl transferase-dUTP nick end labeling (TUNEL) labeling of retinal slices obtained 1 day after ischemia, the presumed time point of the peak apoptotic activity in the HIOP-induced rodent retinal ischemia model.^24^ No TUNEL-labeled cell nuclei were found in untreated retinae, neither of Wt nor of P2Y1R-KO mice (Figure 1d). Between 40 and 50% of the cell nuclei in the INL were found to be TUNEL labeled in the postischemic retinae of Wt and P2Y1R-KO mice (Figure 1e). A similar percentage of cells was TUNEL positive in the ONL of Wt mice, whereas this number was significantly increased in the ONL of P2Y1R-KO (*P*<0.01; Figure 1e).

### Differential retinal gene expression in Wt and P2Y1R-KO mice

**Figure 2:**
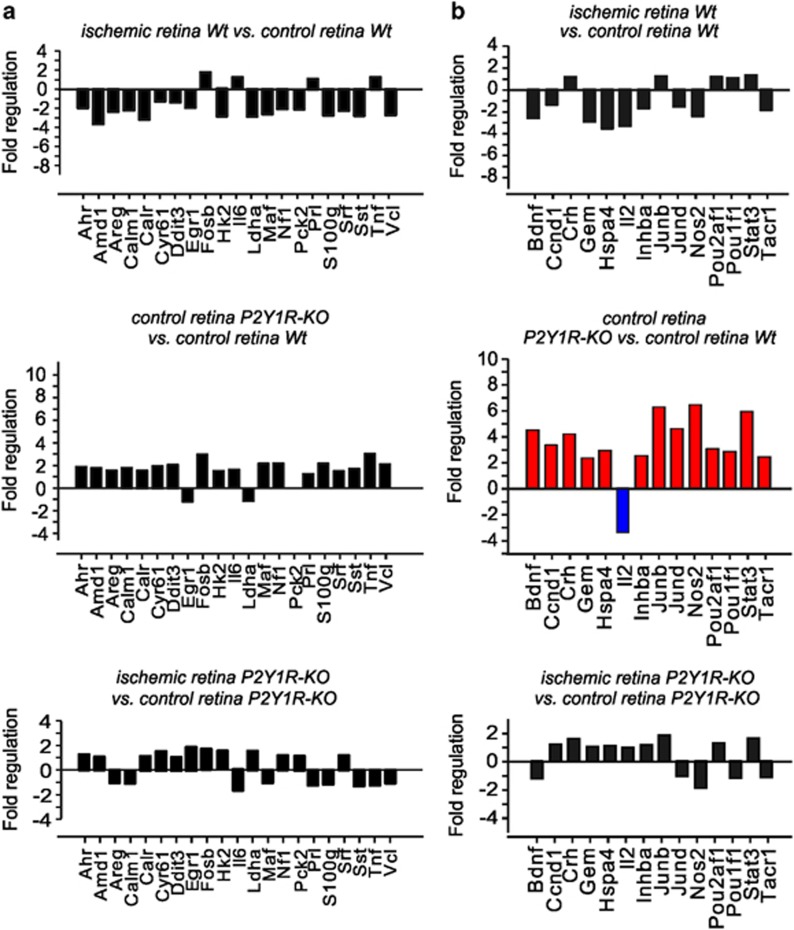
Gene expression profiling in retinae of Wt and P2Y1R-KO mice. The tissues were isolated 7 days after transient retinal ischemia of 90 min and from untreated control eyes from three mice of each genotype. (**a**) Genes that did not show any differences in expression levels according to the analysis using the RT^2^ Profiler PCR Array Data Analysis software. (**b**) Genes are depicted that are differentially expressed in control retinae of Wt and P2Y1R-KO mice. Genes in red were significantly upregulated, genes in blue were significantly downregulated compared with the respective reference group

To assess information about a differential gene expression in control and postischemic retinae as a consequence of P2Y_1_ receptor deficiency, which may explain the described differential degeneration pattern in Wt and P2Y1R-KO mice, we performed a RT^2^ Profiler PCR Array cAMP (3′-5′-cyclic adenosine monophosphate)/Ca^2+^ Signalling PathwayFinder. Expression levels of a total of 84 genes (see also Supplementary Table S1) from retinae 7 days after transient ischemia of 90-min duration were investigated. For 21 genes, no major changes in expression levels were observed — neither if expression levels in postischemic retinae were compared with the respective control eyes, nor if expression levels were compared between Wt and P2Y1R-KO retinae (Figure 2a). We found 14 genes with different expression in the untreated retina of P2Y1R-KO mice compared with Wt retinae; all but one were upregulated (Figure 2b). The expression levels of these genes remained unaltered after ischemia in retinae of Wt and P2Y1R-KO mice. At least five of the upregulated genes are known to be expressed in Müller cells, including the transcription factors *Junb* and *Stat3* (see Supplementary Table, for abbreviations of genes).^25, 26^ A higher expression level of the enzyme, inducible nitric oxide synthase (iNOS; *Nos2*), was also observed (Figure 2b). *Nos2* is known to be expressed in microglial and Müller cells of the ischemic retina.^27, 28^ The higher expression levels of *Junb*, *Nos2*, *Stat3* and *Tacr1* (Figure 2b) may suggest a slight chronic activation of microglia and Müller cells in the P2Y1R-KO retina.

**Figure 3:**
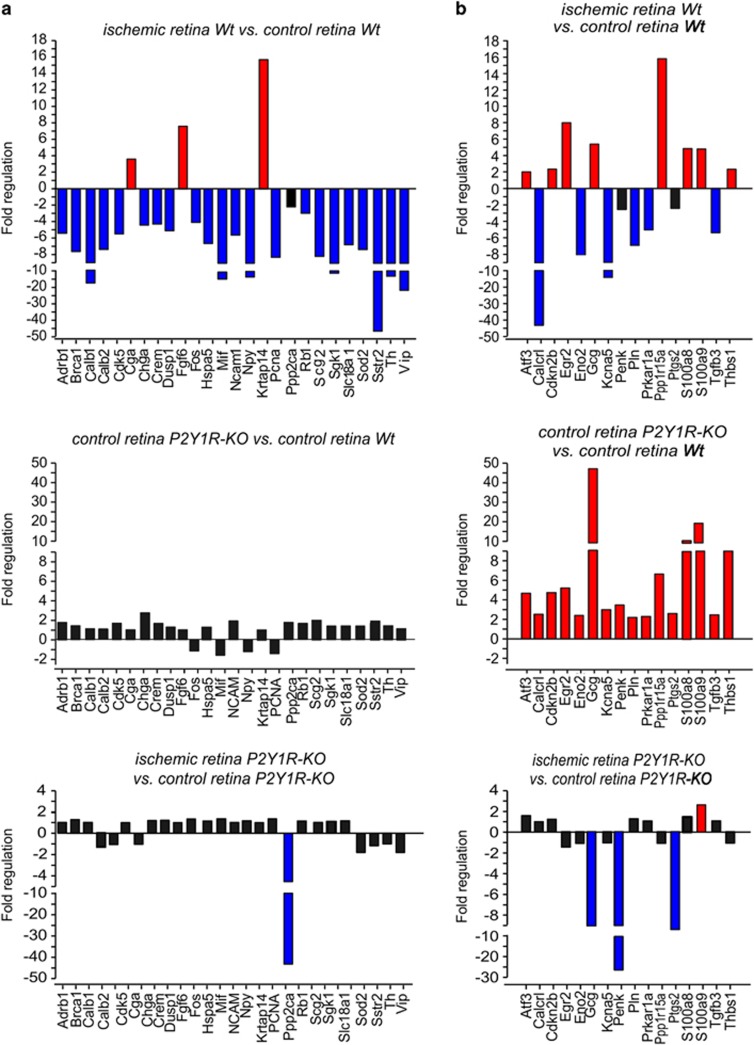
Ischemia-induced alterations in gene expression in retinae of Wt and P2Y1R-KO mice 7 days after 90 min of transient ischemia. Relative expression levels for two groups of genes are shown (**a**) genes that were differentially expressed in the ischemic retinae of Wt animals only and were stable after transient ischemia in retinae of P2Y1R-KO animals, and (**b**) a smaller subset of genes that displays a rather complicated regulation pattern. Genes in red were significantly upregulated, genes in blue significantly downregulated compared with the respective reference group

Transient ischemia of the Wt retina induced alterations in the expression of 24 genes of which 21 were downregulated (Figure 3a). Calbindin (*Calb*) is expressed in amacrine and horizontal cells of the rat retina and was downregulated after retinal ischemia (Figure 3a).^29^ Amacrine cells (ACs) also express neuropeptide Y (*Npy*), vasoactive intestinal polypeptide (*Vip*) and tyrosine hydroxylase (*Th*).^30, 31, 32^ In the postischemic retina of P2Y1R-KO mice, only the expression of *Ppp2ca*, the gene of the *α*-isoform of the catalytic subunit of the protein phosphatase 2 (PP2A), was markedly decreased (Figure 3a). PP2A is present in rod outer segments where it is involved in vesicle trafficking within photoreceptor synapses.^33, 34, 35^

Finally, we found a group of 16 genes that were differentially expressed in control and ischemic retinae of Wt and P2Y1R-KO mice (Figure 3b). The expression levels of these genes were higher in control retinae of P2Y1R-KO mice than in control retinae of Wt mice (Figure 3b). Among these genes are those for transcription factors (*Atf3* and *Egr2*), enzymes (*Eno2*, *Prkar1a*, *Ppp1r15a* and *Ptgs2*), and the neuropeptides glucagon (*Gcg*) and preproenkephalin (*Penk*) (Figure 3b). Both neuropeptides are expressed in ACs.^36, 37^ We found increased expression of the presumable microglial voltage-gated potassium channel Kv1.5 gene (*Kcna5*)^38^ in control retinae of P2Y1R-KO mice compared with Wt retinae (Figure 3b).

Glial cells are one source of thrombospondin-1, a matricellular glycoprotein with manifold functions. It supports neurite formation, has anti-inflammatory effects and prevents angiogenesis in the retina.^39, 40, 41, 42^ Putative downstream signaling mechanisms involve activation of transforming growth factor-*β* (TGF-*β*).^43^ Here we found a remarkably higher level of thrombospondin-1 gene (*Thbs1*) expression together with a slightly upregulated gene expression of TGF-*β*3 (*Tgfb3*) in control P2Y1R-KO retinae if compared with the Wt tissue. In agreement with recent studies,^44^ we found a moderate upregulation of *Thbs1* in postischemic Wt retinae, but the expression level did not reach the level detected in retinae of P2Y1R-KO animals.

### Effects on specific retinal neurons

**Figure 4:**
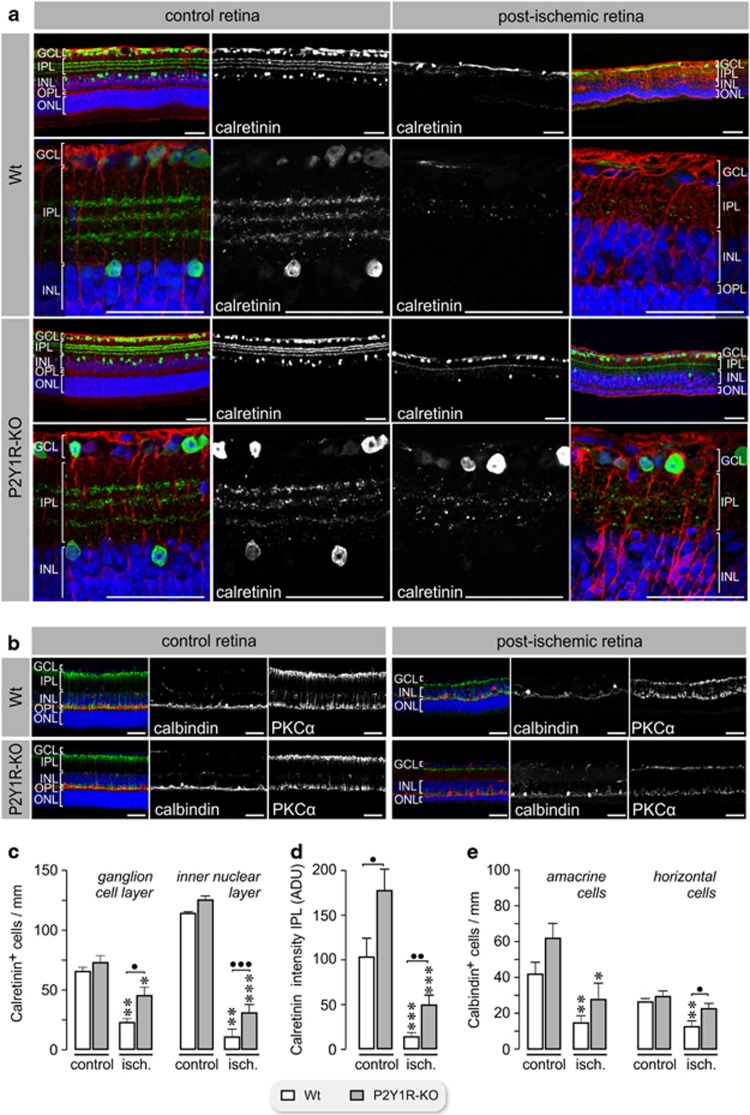
Effects of transient ischemia on retinal neurons 7 days after 90 min of transient ischemia. (**a**) Retinal slices were immunostained for calretinin (green) and CRALBP (red). Cell nuclei were labeled with TO-PRO-3 (blue). Ischemia resulted in a marked reduction of the calretinin staining of distinct cell bodies and a disappearance of the calretinin-labeled bands in the IPL. These effects were more pronounced in the retina of Wt mice than in the P2Y1R-KO retina. (**b**) Calbindin (red) and PKC*α* (green) immunoreactivities in retinal slices. Cell nuclei were labeled with TO-PRO-3 (blue). Postischemic retinae displayed reduced levels of calbindin immunoreactivity in the INL compared with untreated control retinae. The level of PKC*α* immunoreactivity was only slightly different between control and postischemic retinae. (**c**) Mean±S.E.M. number of calretinin-positive cells counted in the INL and the GCL. (**d**) Mean±S.E.M. relative intensity of calretinin immunoreactivity in the IPL, reflecting the calretinin content of AC dendrites. The data were obtained in a 460-*μ*m-wide area. (**e**) Mean±S.E.M. number of calbindin-positive amacrine and horizontal cells. (**c–e**) Data were obtained by analysis of 10–14 central retinal slices derived from 3 to 4 animals. Significant difference to values from the respective untreated control: *P<0.05, **P<0.01, ***P<0.001; ^●^*P*<0.05, ^●●^*P*<0.01, ^●●●^*P*<0.001. Scale bars, 20 *μ*m. OPL, outer plexiform layer

The gene profiling data suggest that various retinal cell types are differently affected by ischemia and absence of P2Y1R. Retinal slices were immunolabeled for the marker proteins calretinin (ganglion cells, ACs),^29, 45^ calbindin (amacrine and horizontal cells), protein kinase C*α* (PKC*α*; bipolar cells) and cellular retinaldehyde-binding protein (CRALBP; Müller cells) to quantify the respective cell types. Calretinin immunostaining revealed positive cell bodies in the GCL and INL and three positive bands in the IPL (Figure 4a). Whereas this staining was markedly reduced after ischemia in the Wt retina in both the GCL and INL, the effect was less prominent in the P2Y1R-KO retina (Figure 4a). The total number of calretinin-positive cells was significantly higher in the postischemic P2Y1R-KO retina compared with values found in Wt mice in both layers (Figure 4c). Of note, we also found major differences in the thickness of the IPL in ischemic retinae of Wt and P2Y1R-KO mice (Figure 1c). As the dendritic tree of retinal ganglion cells and ACs, which seem to be less affected in P2Y1R-KO mice, should considerably contribute to IPL thickness, we determined the overall fluorescence signal of calretinin immunoreactivity in the IPL. In line with the above-mentioned assumption and with the observed superior survival of calretinin-positive cells *per se*, the level of calretinin immunoreactivity in the IPL (Figure 4d) was significantly higher in ischemic retinae of P2Y1R-KO animals than in ischemic retinae of Wt animals.

Calbindin immunoreactivity was observed in ACs (located in the inner part of the INL) and horizontal cells (located in the outer part of the INL) with a tendency of a minor loss of calbindin-labeled cells in the postischemic P2Y1R-KO retina as compared with the retinae of Wt mice (Figures 4b and e). We found no apparent differences in the PKC*α* immunolabeling of bipolar cells (Figure 4b) and in the CRALBP immunostaining of Müller cells (Figure 4a) between the retinae of both mouse strains.

### Müller cell gliosis

**Figure 5:**
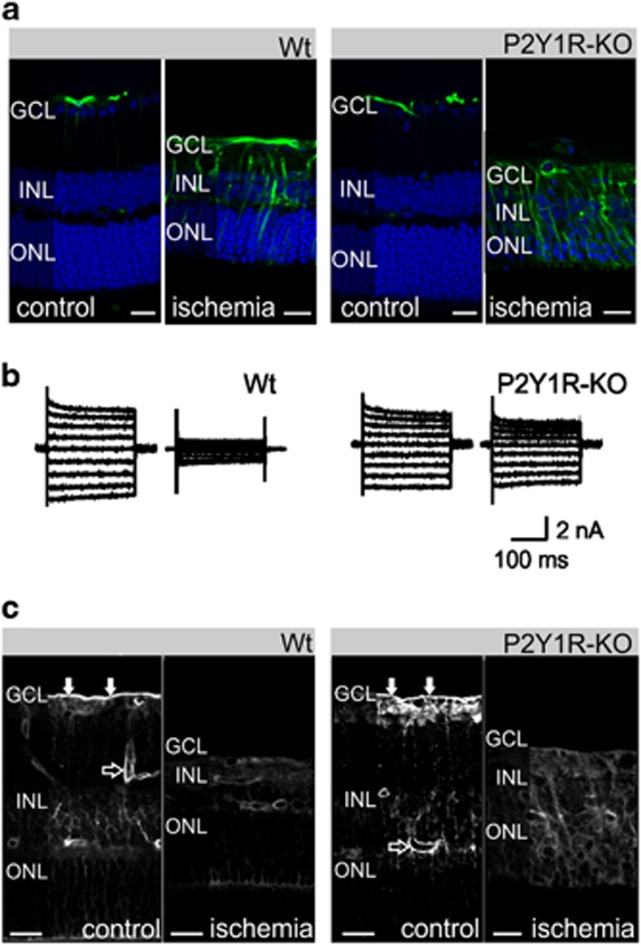
Ischemia-induced Müller cell gliosis. (**a**) GFAP immunoreactivity (green) in slices of retinae isolated 7 days after transient retinal ischemia of 90 min. Labeling of cell nuclei (blue) revealed reduced cell numbers in all nuclear layers. (**b**) Examples of whole-cell K^+^ currents recorded in Müller cells isolated from untreated control retinae (left for each strain) and from retinae that were obtained 7 days after transient retinal ischemia of 90 min (right for each strain). Outward currents are depicted upwardly, inward currents are depicted downwardly. Currents were evoked by 20-mV incremental steps from a holding potential of −80 mV. (**c**) Distribution of Kir4.1 immunoreactivity in slices of control retina and of retinae obtained 7 days after transient retinal ischemia of 90 min. Filled arrows, Kir4.1 labeling of the inner limiting membrane. Unfilled arrow, perivascular Kir4.1 labeling. Scale bars, 20 *μ*m

In the normal retina, the intermediate filament protein, glial fibrillary acidic protein (GFAP), is predominantly expressed by retinal astrocytes rather than by Müller cells; upregulation of GFAP is an early marker of Müller cell gliosis.^46^ We found that Müller cells were devoid of GFAP immunoreactivity in the untreated retinae of Wt and P2Y1R-KO mice (Figure 5a). In contrast, Müller cells in the postischemic retinae of Wt and P2Y1R-KO mice were immunolabeled for GFAP over their entire length.

In line with findings from Hirrlinger *et al.*,^47^ we found that Müller cells of Wt animals, isolated 7 days after transient retinal ischemia for 60 min, displayed a reduction of the Kir currents by approximately 25% as compared with cells from untreated retinae (Table 1). Prolongation of ischemia up to 90 min aggravated this effect resulting in a decrease of the Kir currents in Wt Müller cells by almost 70%. Ischemia also induced an increase in the membrane capacitance of Wt Müller cells (Table 1), suggesting a hypertrophy of the cells.

As P2Y1R signaling was implicated to be involved in gliosis induction,^48^ we investigated the dependence of the ischemic reduction of the Kir currents on purinergic signaling. Surprisingly, cells from untreated P2Y1R-KO eyes displayed a small but significant (*P*<0.05) reduction of the Kir current amplitude by about 10% if compared with cells from untreated Wt retinae (Table 1). Transient ischemia of 60 or 90 min resulted in a Kir current amplitude reduced by 10–20% of the control current in P2Y1R-KO mice (Table 1). This reduction was significantly less pronounced than in cells from Wt animals (Figure 5b; Table 1). The data indicate that deletion of P2Y_1_ attenuates the effect of retinal ischemia-reperfusion on the Kir currents of Müller cells.

We found a slight but significant (*P*<0.01) increase in the membrane capacitance of isolated Müller cells from untreated P2Y1R-KO mice as compared with cells from Wt animals suggesting an increase in the size of Müller cells of P2Y1R-KO animals (Table 1). The membrane capacitance was explicitly increased only in some but not all cases of ischemia, whereas ischemia always induced a decrease of inward current densities (Table 1).

The predominant Kir channel subtype expressed by Müller cells is Kir4.1.^49, 50^ It is concentrated in cell membranes contacting inner limiting membrane and in those surrounding blood vessels. Kir4.1 immunolabeling was similar in retinal slices from untreated Wt and P2Y1R-KO eyes (Figure 5c). Kir4.1 immunoreactivity was more evenly distributed and partially downregulated in the postischemic Wt and P2Y1R-KO retina.

### Microglia activation

In addition to Müller cells, microglial cells were shown to express P2Y_1_.^17, 51^ To assure that observed effects of P2Y_1_R deficiency were largely due to an altered gliotic activation of Müller glia rather than being mediated by a changed activation pattern of microglia, we characterized the latter on the basis of cell numbers and morphological parameters (Supplementary Figure S1) in retinal tissues isolated 1 day after HIOP-induced retinal ischemia for 90 min. We only found minor differences in the characteristics of microglia in the control and postischemic retina of Wt and P2Y1R-KO mice (see Supplementary Information), which do not explain the finding of an altered degeneration pattern in postischemic Wt and P2Y1R-KO retinae.

## Discussion

### Effects of P2Y_1_ deficiency in the control retina

Analysis of the untreated retina revealed significantly less cells in the GCL and INL of P2Y1R-KO compared with Wt mice (Figure 1b). As P2Y_1_ stimulates the proliferation of retinal progenitors,^52^ our data may indicate that P2Y_1_-mediated signaling has a role in the development of the murine retina. Alternatively, and although we did not find TUNEL-positive cells in the control P2Y1R-KO retinae (Figure 1d), slow degenerative processes in the adult P2Y1R-KO retina leading to the reduced cell numbers cannot be excluded. Interestingly, cellular hypertrophy and decreased Kir current amplitudes (Table 1), but no elevation in GFAP expression, suggest a low-level Müller cell reactivity in the P2Y1R-KO retina. Gene expression profiling revealed enhanced expression levels of *Nos2*, *Stat3*, *Junb* and *Thbs1* in untreated P2Y1R-KO retinae (Figures 2b and 3b) whose upregulation has been associated with Müller cell gliosis.^25, 26, 28^ For example, Müller cells produce thrombospondin-1 under ischemic conditions.^44^ Thrombospondin-1 activates latent TGF-*β*.^43^ Accordingly, we also detected an enhanced expression of TGF-*β*3 and of its downstream target inhibin *β*-A (*Inhba*)^53^ in P2Y1R-KO control retinae (Figures 2b and 3b). Upregulation of *S100a8/S100a9*, as found in P2Y1R-KO retinae (Figure 3b), has been associated with microglia activation.^54, 55^ However, we found only slight differences in the microglial morphology between P2Y1R-KO and Wt retinae (Supplementary Figure S1). Possibly, thrombospondin-1 counterbalances microglia activation in P2Y1R-KO animals.^37^ It remains to be determined whether the low-level glial activation in the P2Y1R-KO retina reflects degenerative processes or alterations of glial function without significant effects on retinal integrity.

### Ischemic retinal degeneration

Significantly less photoreceptors survived in P2Y1R-KO mice than in Wt retinae (Figure 1b). The TUNEL assay, revealing more apoptotic cells in the ONL of P2Y1R-KO than in Wt mice, confirmed this finding (Figure 1e). Interestingly, the IPL in postischemic P2Y1R-KO retinae was thicker than in Wt mice, despite similar cell numbers in the GCL and INL (Figure 1c), probably indicating a better preservation of neurites in the IPL of P2Y1R-KO mice. Gene expression profiling points to ACs as candidates displaying increased survival in the P2Y_1_-deficient postischemic retina. We found that 12 genes that are primarily expressed in ACs^29, 30, 31, 32^ were downregulated in the postischemic Wt but stably expressed in the postischemic P2Y1R-KO retinae (Figure 3a). These data were confirmed by immunolabelings showing higher levels of AC (calretinin, calbindin), but also ganglion cell (calretinin) markers in postischemic P2Y1R-KO retinae indicating a better survival of these cell types (Figure 4). Both cell types are highly susceptible to ischemic damage.^27, 56^ We found that only 15% of putative ACs survived in postischemic Wt retinae, whereas the mean overall reduction of retinal cells was approximately 50% (Figures 1b and 4c).

### Effects of P2Y_1_ deficiency on ischemia-induced Müller cell gliosis

Transient retinal ischemia resulted in a reduction of the Kir current amplitude, which was significantly weaker in P2Y1R-KO Müller cells than in Wt cells (Table 1). Therefore, we concluded that P2Y_1_ signaling is involved in the regulation of the Kir channel expression during gliotic Müller cell activation. This is in agreement with previous studies showing that P2Y_1_ signaling has a role in gliosis induction in the brain.^13, 48^

Using gene expression profiling, we found that several genes rather specifically expressed in Müller cells (e.g., *Adrb1*, *Calcrl*, *Chga*, *Mif* and *TGFβ3*) were differently regulated by ischemia in P2Y1R-KO retinae compared with Wt retinae (Figure 3). A differential Müller cell degeneration as a reason for the downregulation of Müller cell-specific genes in Wt retinae appears unlikely, because the overall appearance of Müller cells was similar in the postischemic Wt and P2Y1R-KO retinae. Instead, we assume that this downregulation is depending on P2Y_1_-mediated signaling.

### Impact of purinergic signaling on glial support of neuronal survival

The low-level activation of Müller and microglial cells in the untreated retina of P2Y1R-KO mice may provide a preconditioning to pathological events like ischemia and, thus, may be protective for inner retinal neurons such as ACs (Figure 4). Preconditioning, for example, by brief ischemia, has neuroprotective effects in the retina^57, 58^ and typically involves an upregulation of enzymes involved in nitric oxide formation, such as iNOS (*Nos2*).^59^ We found a higher expression level of *Nos2* in the retina of P2Y1R-KO mice as compared with Wt retina (Figure 2b), however, we did not study the functional role of iNOS in more detail. Therefore, it remains to be determined in future whether the glial activation in the retina of P2Y1R-KO mice provides preconditioning.

**Figure 6:**
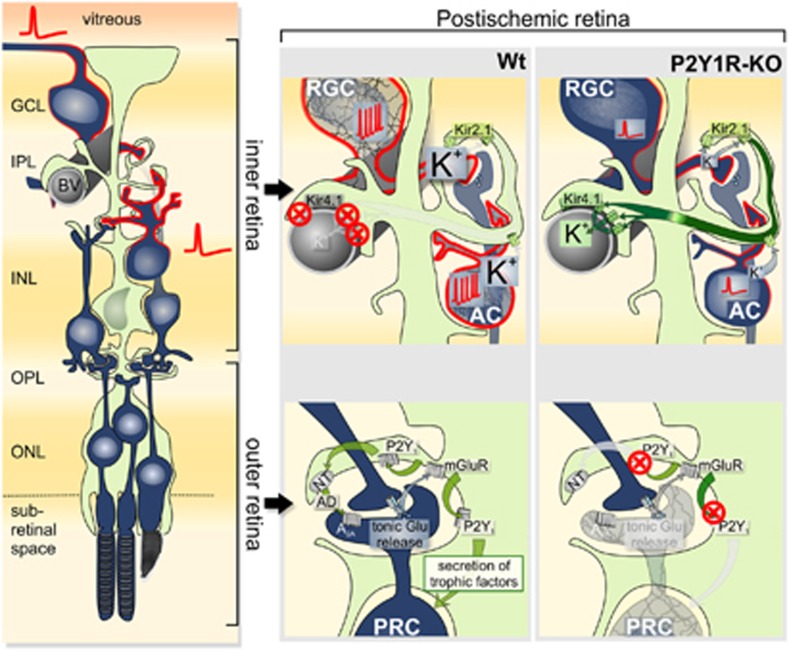
Artist's view on the putative janus-faced effect of P2Y_1_ deficiency in the postischemic retina. Left: schematic drawing of a Müller cell (light green) and its associated neurons (dark blue). Right: downregulation of Kir4.1 in Wt mice prevents Müller cells from fulfilling their important function to maintain the retinal potassium homeostasis by transcellular clearance of the latter from the extracellular space into bigger fluid filled compartments such as the blood system. This may lead to cytotoxic neuronal hyperexcitation especially of cells generating action potentials such as retinal ganglion cells and subtypes of ACs (indicated by red membrane) in the inner retina. In contrast, photoreceptors in the outer retina appear to benefit from functional P2Y1R, probably due to P2Y_1_-mediated release of neuroprotective substances like AD or other trophic factors. In P2Y1R-KO mice the stable Kir4.1 expression appears to allow a better survival of neurons in the inner retina, while the lack of P2Y_1_R considerably disrupts the essential support of photoreceptors by Müller glia. BV, blood vessel; mGluR, metabotropic Glu receptor; NT, nucleoside transporter; OPL, outer plexiform layer; PRC, photoreceptor cell; RGC, retinal ganglion cell

In contrast to this ‘baseline' glial activation, some aspects of reactive Müller cell gliosis, including the decrease in Kir currents (Table 1), are alleviated in the retina of P2Y1R-KO mice if compared with the Wt. This suggests that P2Y_1_ signaling is involved in the regulation of distinct aspects of Müller cell gliosis, particularly, the expression level of Kir4.1 channels. The rather high potassium conductance of Müller cells and the unaltered or increased glial expression of neurotrophic and anti-inflammatory factors such as TGF-*β*3 (*Tgfb3*), macrophage migration inhibitory factor (*Mif*) and thrombospondin-1 (*Thbs1*) (Figure 3) presumably account for the improved survival of some neuronal subtypes in the inner retina of P2Y1R-KO animals (Figure 6). The maintained high potassium conductance of Müller cells in the postischemic P2Y1R-KO retina may stabilize the ion and volume homeostasis, and, thereby, may prevent a cytotoxic hyperexcitation of the already energy-deprived neurons of the inner retina being highly sensitive to hyperexcitation associated with ischemia (Figure 6).^27, 56, 60^

A different scenario was observed in the outer retina. An increased photoreceptor cell loss in the postischemic P2Y1R-KO compared with the Wt retinae (Figure 1) suggests that P2Y_1_-mediated signaling has a protective effect on photoreceptors in ischemia. Müller cells support photoreceptors by delivery of lactate and neurotrophic factors.^61, 62^ Selective ablation of Müller cells results in photoreceptor loss.^63, 64^ It remains to be clarified in future studies, which of these putatively involved mechanisms are affected in the postischemic P2Y1R-KO retina. Further mechanisms, for example, the regulation of the extracellular space volume,^65^ may contribute to the glial support of photoreceptor survival (Figure 6). One major alteration in Müller cell physiology in P2Y1R-KO mice is the impaired capability to maintain cellular volume control under hypoosmotic conditions.^16^ The latter involves a signaling cascade induced by an activation of metabotropic Glu and P2Y_1_ receptors, and glial release of adenosine 5′-triphosphate and adenosine (AD).^14^ In the outer plexiform layer, this signaling cascade may be continuously activated by the constant release of Glu from photoreceptor terminals.^66^ Interruption of this feedback loop in P2Y1R-KO retinae may contribute to photoreceptor degeneration because of the impaired Glu-induced release of the neuroprotectant AD from Müller cells (Figure 6).^67^

Anti-purinergic agents were suggested to serve as therapeutics for the treatment of ischemic disorders in neural tissues. The present results of differential effects of P2Y_1_ deficiency on the survival of neuronal subtypes in the ischemic retina call the use of such agents into question. This is consistent with conflicting results regarding a potential protective effect of P2Y signaling in ischemia published by others. P2Y_1_ antagonists applied in a rat model of cerebral ischemia reduced the infarct volume and improved motor function recovery.^68^ In contrast, neuroprotective effects of P2Y_1_-mediated signaling in brain astrocytes under various pathological conditions have been reported.^69, 70, 71^ Further research is required to determine — and to separate — the beneficial and detrimental effects of P2Y_1_-mediated signaling in the ischemic brain and retina.

## Materials and Methods

### Materials

Papain was from Roche (Mannheim, Germany). All other substances used were from Sigma-Aldrich (Taufkirchen, Germany), unless stated otherwise. The following primary antibodies were used: rabbit anti-Kir4.1 (1 : 200; Sigma-Aldrich), mouse anti-GFAP (1 : 200; G-A-5 clone, Sigma-Aldrich), rabbit anti-ionized calcium binding adaptor molecule 1 (Iba1; 1 : 500, Wako, Neuss, Germany), goat anti-calretinin (1 : 500, Swant, Marly, Switzerland), mouse anti-calbindin (1 : 400, Swant), rabbit anti-PKC*α* (1 : 300, Santa Cruz Biotechnology, Heidelberg, Germany), rabbit anti-CRALBP (1 : 300, Santa Cruz Biotechnology) and mouse anti-glutamine synthetase (1 : 1000, Merck Millipore, Darmstadt, Germany). The following secondary antibodies were used: Cy5-conjugated donkey anti-goat, Cy3-conjugated donkey anti-rabbit, Cy2-conjugated donkey anti-mouse, Cy3-conjugated goat anti-rabbit and Cy2-conjugated goat anti-mouse. All secondary antibodies were applied in a 1 : 200 dilution and were obtained from Dianova (Hamburg, Germany). The apoptosis rate was detected using the *in situ* cell death detection kit, tetramethylrhodamine red (Roche).

### Animals

All experiments were done in accordance with the European Communities Council Directive 86/609/EEC, and were approved by the local authorities. Animals were maintained with free access to water and food in an air-conditioned room on a 12-h light–dark cycle. Adult (2–6 months old) mice deficient in the nucleotide receptor P2Y_1_ (P2Y1R-KO; 129Sv background) were characterized as described.^72^ Briefly, for genotyping of the P2Y1R-KO mice and wild-type (Wt) littermate controls, the genomic region of the P2Y_1_ receptor was characterized. PCR analysis was made with the following primers: *knockout*, *common,* 5′-GCAGTGTTTGGGGTCAGAAT-3′, *neo*, 5′-GGGGAACTTCCTGACTAGGG-3′ *Wt*, *common*, *5*′-GCAGTGTTTGGGGTCAGAAT-3′, *Wt*, 5′-AACATACGCTGCAAGGCTCT-3′. Age- and weight-matched littermate Wt controls were used.

### Retinal ischemia

Transient retinal ischemia was induced in one eye by the HIOP method. The other eye remained untreated as internal control. Anesthesia was induced with ketamine (100 mg/kg body weight, intraperitoneal (i.p.); Ratiopharm, Ulm, Germany), xylazine (5 mg/kg, i.p.; Bayer Vital, Leverkusen, Germany) and atropine sulfate (100 mg/kg, i.p.; Braun, Melsungen; Germany). The anterior chamber of the test eye of anesthetized mice was cannulated from the pars plana with a 30-gauge infusion needle, connected to a saline bottle. The intraocular pressure was increased to 160 mm Hg for 60 or 90 min by elevating the bottle. After removing the needle, the animals survived for 1 or 7 days and, subsequently, were killed with carbon dioxide.

### Preparation of isolated Müller cells

Isolated retinae were incubated in papain (0.2 mg/ml)-containing Ca^2+^-/Mg^2+^-free phosphate-buffered saline, pH 7.4, for 30 min at 37 °C, followed by several washing steps with saline. After short incubation in saline supplemented with deoxyribocnuclease I (200 U/ml), the tissue pieces were triturated by a 1-ml pipette tip, to obtain isolated retinal cells. The cells were stored at 4 °C in serum-free minimum essential medium until use within 4 h after cell isolation. Müller cells were identified in the cell suspensions according to their characteristic morphology.

### Whole-cell patch-clamp records of isolated Müller cells

The whole-cell currents of freshly isolated Müller cells were recorded at room temperature using the Axopatch 200A amplifier (Axon Instruments, Foster City, CA, USA) and the ISO-2 computer program (MFK, Niedernhausen, Germany). The signals were low-pass filtered at 1, 2 or 6 kHz (eight-pole Bessel filter) and digitized at 5, 10 or 30 kHz, respectively, using a 12-bit A/D converter. Patch pipettes were pulled from borosilicate glass (Science Products, Hofheim, Germany) and had resistances between 4 and 6 MΩ when filled with a solution containing (mM) 10 NaCl, 130 KCl, 1 CaCl_2_, 2 MgCl_2_, 10 ethylene glycol tetraacetic acid and 10 4-(2-hydroxyethyl)-1-piperazineethanesulfonic acid (HEPES), adjusted to pH 7.1 with tris(hydroxymethyl)aminomethane (Tris). The recording chamber was continuously perfused with extracellular solution that contained (mM) 135 NaCl, 3 KCl, 2 CaCl_2_, 1 MgCl_2_, 1 Na_2_HPO_4_, 10 HEPES and 11 glucose, adjusted to pH 7.4 with Tris. To evoke potassium currents, de- and hyperpolarizing voltage steps of 250 ms duration, with increments of 10 mV, were applied from a holding potential of −80 mV. The amplitude of the steady-state Kir currents was measured at the end of the 250-ms voltage step from −80 to −140 mV. The membrane capacitance of the cells was measured by the integral of the uncompensated capacitive artifact (filtered at 6 kHz) evoked by a 10-mV voltage step in the presence of extracellular BaCl_2_ (1 mM). Current densities were calculated by dividing inward current amplitudes evoked by 60 mV hyperpolarization by the membrane capacitance. The resting membrane potential was measured in the current-clamp mode.

### Histological staining and immunohistochemistry

Isolated retinae were fixed in 4% paraformaldehyde for 2 h. After several washing steps in buffered saline, the tissues were embedded in saline containing 3% agarose (w/v), and 70-*μ*m thick slices were cut by using a vibratome. The slices were incubated in 5% normal goat serum plus 0.3% Triton X-100 plus 1.0% dimethyl sulfoxide in saline for 2 h at room temperature and, subsequently, in the primary antibodies overnight at 4 °C. After washing in 1% bovine serum albumin, the secondary antibodies were applied for 2 h at room temperature. Cell nuclei were labeled with TO-PRO-3 (1 : 1000; Life Technologies, Carlsbad, CA, USA). Control slices were stained without the primary antibody; no unspecific labeling was observed following incubation with the secondary antibody alone (data not shown). Images were taken with the LSM 510 Meta (Zeiss, Oberkochen, Germany). The number of cell nuclei in different retinal layers was counted in a 100-*μ*m-wide area close to the optic nerve head (optical slice thickness, 1.5 *μ*m; Figure 1a).

Iba1-immunolabeling revealed microglial cells in paraformaldehyde-fixed retinal whole-mount preparations. Z-stacks created at the laser scanning microscope (LSM) were used to quantify microglia properties using the software of the LSM. First, the number of cells was counted in different retinal layers in an area of 325 × 325 *μ*m. Second, the area occupied by individual cells was determined by recording the area confined by the most distal ends of microglial processes. Third, the number of branching points per cell was detected. A branching point was defined as a spot on a microglial process where this process is divided into two parts of >2 *μ*m length. Finally, the total area of the soma was calculated.

The TUNEL assay was performed according to the manufacturer's protocol (Roche). To assure good accessibility for the components of the assay to the tissue, we subjected free floating retinal slices to a brief microwave treatment in citrate buffer (pH 6.0, 0.1 M). After washing with buffered saline and tissue permeabilization for 10 min in 4% Triton X-100, the slices were incubated for 90 min in the labeling solution. Afterward, the slices were counterstained with TO-PRO-3 and mounted onto slides.

### Real-time PCR

Total RNA was prepared from control and postischemic (after 7 days survival) retinae of Wt and P2Y1R-KO mice (*n*=3 each) with RNAeasy Mini Kit (Qiagen, Hilden, Germany) according to the manufacturer's instructions. The quality of the RNA was controlled by agarose gel electrophoresis. A NanoDrop spectrophotometer was used to measure RNA concentration. The A260/A280 ratio of the optical density was >1.9 in all samples, indicating highly purified RNA. cDNA was synthesized with 300 ng RNA for each sample using the RT^2^ First Strand Kit (Qiagen). The investigation of gene expression was carried out using the RT^2^ Profiler PCR Array cAMP/Ca^2+^ Signalling PathwayFinder (Qiagen; Supplementary Table S1). Real-time PCR was performed following the manufacturer's instruction using a Rotor-Gene 6000 cycler (Corbett Research, Sydney, Australia) and RT^2^ SYBR Green/ROX qPCR Master Mix (Qiagen). Changes in gene expression levels were calculated according to the ΔΔCt method using the web based RT^2^ Profiler PCR Array Data Analysis software (http://pcrdataanalysis.sabiosciences.com/pcr/ arrayanalysis.php).

### Statistics

Data are expressed as mean±S.E.M. or S.D. (patch-clamp data). Statistical analysis was made using Prism (Graphpad Software, San Diego, CA, USA); significance was determined by the non-parametric Mann–Whitney *U-*test.

## Figures and Tables

**Table 1:** Electrophysiological properties of murine Müller cells

	**Wt**			**P2Y1R-KO**		
	**Untreated**	**HIOP 60** **min**	**HIOP 90** **min**	**Untreated**	**HIOP 60** **min**	**HIOP 90** **min**
Inward current amplitude (pA)	3082±606	2237±1069***	989±680***	2753±761^○^	2238±803**	2358±975*^,○○○^
Relative inward currents (%)	100±19	75±35***	32±22***	100±20	90±48	82±37**^,○○○^
Membrane potential (mV)	−86±4	−85±3	−78±17**	−85±4	−85±5	−86±5^○^
Membrane capacitance (pF)	46±14	67±9***	53±15	56±13^○○^	70±16***	64±10**^,○○^
Current density (pA/pF)	74±25	34±15***	29±12***	53±21^○○○^	36±17***	37±15***
numbers	9 mice	6 mice	3 mice	11 mice	6 mice	5 mice
	42 cells	31 cells	19 cells	61 cells	34 cells	29 cells

The cells were isolated 7 days after HIOP-induced retinal ischemia. For the calculation of the relative inward currents, the mean current amplitude recorded from cells of the untreated control eye was set to 100% for each mouse, and the relative value for each cell from the contralateral postischemic eye was calculated. *P<0.05, **P<0.01, ***P<0.001 compared with the respective value of the untreated eye from animals of the same strain. ^○^*P*<0.05, ^○○^*P*<0.01, ^○○○^*P*<0.001 compared with the respective value from Wt mice. Data are given as mean±S.D.

## Abbreviations

AC: amacrine cell
AD: adenosine
cAMP: 3′-5′-cyclic adenosine monophosphate
CRALBP: cellular retinaldehyde-binding protein
Cy: carbocyanine
GCL: ganglion cell layer
GFAP: glial fibrillary acidic protein
Glu: glutamate
HEPES: 4-(2-hydroxyethyl)-1-piperazineethanesulfonic acid
HIOP: high intraocular pressure
Iba1: ionized calcium binding adaptor molecule 1
INL: inner nuclear layer
iNOS: inducible nitric oxide synthase
i.p.: intraperitoneal
IPL: inner plexiform layer
Kir: inwardly rectifying potassium channel
LSM: laser scanning microscope
ONL: outer nuclear layer
PKC*α*: protein kinase C*α*
P2Y1R: P2Y_1_ receptor
P2Y1R-KO: P2Y_1_ receptor-deficient
TGF-*β*3: transforming growth factor-*β*3
Tris: tris(hydroxymethyl)aminomethane
TUNEL: terminal deoxynucleotidyl transferase-dUTP nick end labeling
Wt: wild type
