# Supplementary material for: Differential effects of P2Y1 deletion on glial activation and survival of photoreceptors and amacrine cells in the ischemic mouse retina
Source: Cell Death Dis. 2014 Jul 31;5(7):e1353–. doi: 10.1038/cddis.2014.317 (PMC4123106; doi:10.1038/cddis.2014.317)
Supplement: Supplementary Information [file cddis2014317x1.doc]

**Supplement**

**Tab. S1 Abbreviations of genes in figure 2 and 3.**

| Refseq | Symbol | Description |
| --- | --- | --- |
| NM_007419 | Adrb1 | Adrenergic receptor, beta 1 |
| NM_007498 | Atf3 | Activating transcription factor 3 |
| NM_007540 | Bdnf | Brain derived neurotrophic factor |
| NM_009764 | Brca1 | Breast cancer 1 |
| NM_009788 | Calb1 | Calbindin 1 |
| NM_007586 | Calb2 | Calbindin 2 |
| NM_018782 | Calcrl | Calcitonin receptor-like |
| NM_007631 | Ccnd1 | Cyclin D1 |
| NM_007668 | Cdk5 | Cyclin-dependent kinase 5 |
| NM_007670 | Cdkn2b | Cyclin-dependent kinase inhibitor 2B (p15, inhibits CDK4) |
| NM_009889 | Cga | Glycoprotein hormones, alpha subunit |
| NM_007693 | Chga | Chromogranin A |
| NM_013498 | Crem | CAMP responsive element modulator |
| NM_205769 | Crh | Corticotropin releasing hormone |
| NM_013642 | Dusp1 | Dual specificity phosphatase 1 |
| NM_010118 | Egr2 | Early growth response 2 |
| NM_013509 | Eno2 | Enolase 2, gamma neuronal |
| NM_010204 | Fgf6 | Fibroblast growth factor 6 |
| NM_010234 | Fos | FBJ osteosarcoma oncogene |
| NM_008100 | Gcg | Glucagon |
| NM_010276 | Gem | GTP binding protein (gene overexpressed in skeletal muscle) |
| NM_008300 | Hspa4 | Heat shock protein 4 |
| NM_022310 | Hspa5 | Heat shock protein 5 |
| NM_008366 | Il2 | Interleukin 2 |
| NM_008380 | Inhba | Inhibin beta-A |
| NM_008416 | Junb | Jun-B oncogene |
| NM_145983 | Kcna5 | Potassium voltage-gated channel, shaker-related subfamily, member 5 |
| NM_013707 | Krtap14 | Keratin associated protein 14 |
| NM_010798 | Mif | Macrophage migration inhibitory factor |
| NM_010875 | Ncam1 | Neural cell adhesion molecule 1 |
| NM_010927 | Nos2 | Nitric oxide synthase 2, inducible |
| NM_023456 | Npy | Neuropeptide Y |
| NM_011045 | Pcna | Proliferating cell nuclear antigen |
| NM_001002927 | Penk | Preproenkephalin |
| NM_023129 | Pln | Phospholamban |
| NM_008849 | Pou1f1 | POU domain, class 1, transcription factor 1 |
| NM_011136 | Pou2af1 | POU domain, class 2, associating factor 1 |
| NM_008654 | Ppp1r15a | Protein phosphatase 1, regulatory (inhibitor) subunit 15A |
| NM_019411 | Ppp2ca | Protein phosphatase 2 (formerly 2A), catalytic subunit, alpha isoform |
| NM_021880 | Prkar1a | Protein kinase, cAMP dependent regulatory, type I, alpha |
| NM_011198 | Ptgs2 | Prostaglandin-endoperoxide synthase 2 |
| NM_009029 | Rb1 | Retinoblastoma 1 |
| NM_013650 | S100a8 | S100 calcium binding protein A8 (calgranulin A) |
| NM_009114 | S100a9 | S100 calcium binding protein A9 (calgranulin B) |
| NM_009129 | Scg2 | Secretogranin II |
| NM_011361 | Sgk1 | Serum/glucocorticoid regulated kinase 1 |
| NM_153054 | Slc18a1 | Solute carrier family 18 (vesicular monoamine), member 1 |
| NM_013671 | Sod2 | Superoxide dismutase 2, mitochondrial |
| NM_009217 | Sstr2 | Somatostatin receptor 2 |
| NM_011486 | Stat3 | Signal transducer and activator of transcription 3 |
| NM_009313 | Tacr1 | Tachykinin receptor 1 |
| NM_009368 | Tgfb3 | Transforming growth factor, beta 3 |
| NM_009377 | Th | Tyrosine hydroxylase |
| NM_011580 | Thbs1 | Thrombospondin 1 |
| NM_011702 | Vip | Vasoactive intestinal polypeptide |

**Characterization of microglial morphology and cell numbers**

We counted microglial cells in the GCL/IPL and in the OPL. The number of microglial cells in the GCL/IPL of P2Y1R-KO mice was slightly but significantly smaller than that of the Wt (Fig. S1 C). However, because of a higher number of microglial cells in the OPL of P2Y1R-KO mice (Fig. S1 C), the total number of retinal microglial cells in the untreated retina was not different between Wt and P2Y1R-KO mice. These data suggest that lack of P2Y_1_ receptors results in a minor redistribution of microglial cells between different retinal layers. One day after transient retinal ischemia of 90 min, there was a significant increase of the number of microglial cells in the GCL/IPL and a decrease in the OPL in the retina of Wt and P2Y1R-KO mice, compared to the untreated controls (Fig. S1 C).

The area occupied by individual microglia cells in the GCL/IPL and OPL of histological sections was slightly smaller in P2Y1R-KO mice compared to those of Wt microglia (Fig. S1 C). Retinal ischemia significantly (P<0.001) decreased the average area occupied by the processes of a cell (‘dendritic domain’; cf. Fig. S1 B) from 4000-6000 to about 2000 µm² in both mice strains (Fig. S1 C). The number of branchings per microglial cell was generally higher in cells residing in the OPL than in cells located in the GCL/IPL (Fig. S1 C). Cells in the GCL/IPL of P2Y1R-KO had fewer branching points than Wt cells (Fig. S1 C). The number of branching points decreased significantly (P<0.001) in microglial cells in postischemic retinae of Wt and P2Y1R-KO mice (Fig. S1 C). Microglial cells in the OPL of P2Y1R-KO mice displayed significantly (P<0.01) smaller soma areas than cells of Wt animals (Fig. S1 C). Retinal ischemia induced significant (P<0.001) increases in the soma area of microglial cells in both groups investigated compared to the untreated controls (Fig. S1 C). In tissues of P2Y1R-KO animals, the soma area increased significantly (P<0.05) more in cells in the GCL/IPL, and less in cells in the OPL, than in the Wt (Fig. S1 C).


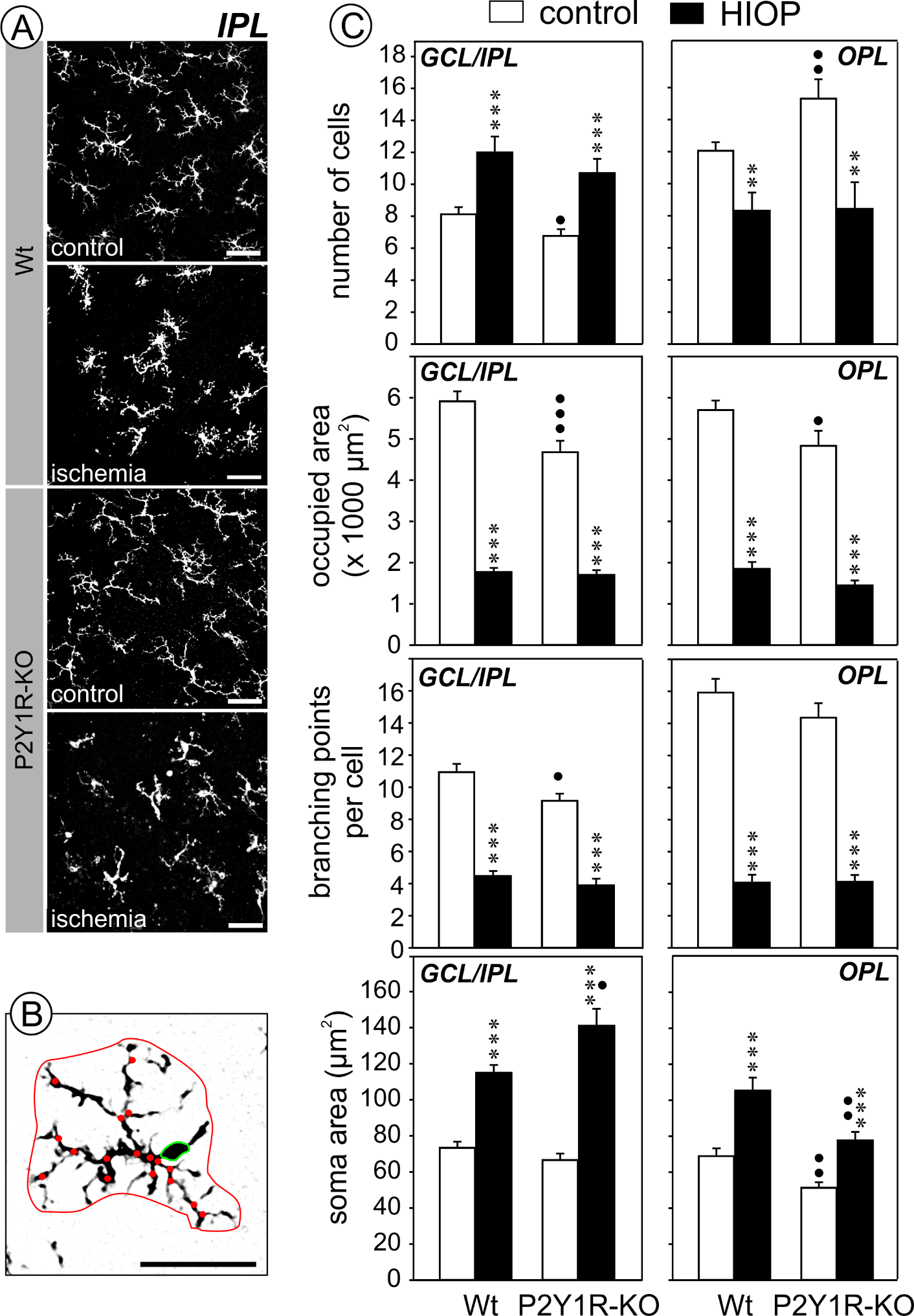


**Fig. S1. Ischemia-induced microglia activation in the retinae of Wt and P2Y1R-KO mice.** The tissues were isolated 1 d after HIOP-induced retinal ischemia of 90 min. **A.** Representative z-stack projection (out of 12-17 optical slices with a thickness of 1.5 µm) of microglia labeled with Iba-1 in the inner plexiform layer (IPL) in control and ischemic retinae of Wt and P2Y1R-KO animals. Activated microglial cells in ischemic retinae displayed shorter and less ramified processes than cells in control retinae and thus occupied smaller areas. Their soma area enlarged compared to cells from control retinae. **B.** Different morphometric parameters were defined for a single microglial cell. The area occupied by the microglia was defined as the tissue reached by the maximal extensions of their longest processes (*red line*). Only those branching points (*red circles*) were included which gave rise to at least two new side processes with at least one additional branching point. The *green circle* depicts the measured soma area. Scale bars, 50 µm. **C.** Mean ± SEM number of microglial cells and morphological parameters of microglial cells. Significant difference to values from the respective untreated control: ***P*<0.01, ****P*<0.001. Significant difference to the value of the Wt: ● *P*<0.05, ●● *P*<0.01, ●●● *P*<0.001. Control and postischemic retinal wholemounts from 4 animals were scanned at four areas 200 µm from the optic nerve head yielding an n=10-14 of scans in which cell numbers were determined. Bars displaying values for occupied area, branching and soma area contain values from 24-44 cells out the scans from 4 animals.
